# Maintaining quality of life and care for cancer survivors experiencing disaster disruptions: a review of the literature

**DOI:** 10.1186/s12885-023-11191-9

**Published:** 2023-07-26

**Authors:** Yoshiko Kitamura, Hisao Nakai

**Affiliations:** 1 School of Nursing, Kanazawa Medical University, 1-1 Uchinada, Kahoku, Ishikawa 920-0265 Japan; 2 Faculty of Nursing, University of Kochi, 2751-1 Ike, Kochi, 781-8515 Japan

**Keywords:** Cancer, Disaster, Health services, Survivorship, Treatment delay, Quality of life

## Abstract

Disasters caused by natural phenomena are increasing in frequency and devastation. The growing number of cancer survivors constitute a vulnerable population in their need for continuous and high-level care, a vulnerability that is exacerbated in the event of disasters. Although the evidence base on the needs of cancer survivors is growing, little is known about cancer care in disaster settings. Therefore, we prepared a narrative literature review that outlines existing evidence, identifies knowledge gaps, and clarifies key concepts that are central to the burgeoning area of research into the quality of care for cancer survivors through disasters. As the preponderance of available evidence stresses the importance of careful disaster planning for maintaining care services, this review also provides guidance in developing plans for how to proceed during, and in the aftermath of, disasters.

## Background

Disasters caused by natural phenomena are increasing in frequency and devastation, with three times as many occurring in the first decade of the twenty-first century as in the 1980s [1]. In 2021, according to the United Nations Centre for Research on the Epidemiology of Disasters, a total of 432 disasters related to natural hazards occurred worldwide (not including the COVID-19 pandemic), affecting 101.8 million people [2]. The effects of these disasters may disproportionately affect vulnerable populations, such as cancer survivors [3].

For the purposes of this review, “disasters” will be considered to include events that may be broadly categorized as geophysical, climate-related (hydro-meteorologic), or biologic. These first two categories include floods, storms, extreme temperature events, wildfires, earthquakes, and volcanic eruptions, while biologic events are exemplified by the COVID-19 pandemic. Manmade disaster events such as war or pollution/radiation events are of a different category, but the lessons learned from disaster medicine experience are nonetheless applicable in most cases, regardless of the source. Among their many effects, disasters of all categories create disruptions in cancer care and other chronic care. A study by Ozaki et al. [4] describing the effects of the 2011 tsunami in Japan on breast cancer treatment delineates many factors leading to care disruptions and long-term psychosocial effects, including the closure of medical institutions, a decrease in available healthcare providers, and a lack of coordinated cancer management or social support. Similarly, care disruptions resulting from the COVID-19 pandemic are a current issue that highlights the repercussions of an unanticipated crisis.

The scope of this problem is sufficiently serious that it has been taken up by the United Nations Office for Disaster Risk Reduction in the form of The Sendai Framework for Disaster Risk Reduction 2015–2030 [5, 6], which outlines four action priorities to reduce disaster risk, namely (i) understanding disaster risk; (ii) strengthening disaster risk governance to manage such events; (iii) investing in disaster reduction for resilience; and (iv) enhancing disaster preparedness for an effective response, and ongoing recovery, rehabilitation, and reconstruction.

As cancer care has evolved from purely palliative care in the early twentieth century, the definition of a cancer survivor has likewise evolved and is now acknowledged to include people with cancer from the time of diagnosis through the initial treatment period and chronic or intermittent disease until the end of life. There are, of course, differences in needs between patients undergoing active treatment and those long-term survivors for whom treatment is complete. Yet for all cancer survivors regardless of their phase of survivorship, quality of life in cancer care is an important concern. Cancer survivors are a vulnerable population in their need for steady and high-level care.

The global cancer burden is expected to be 28.4 million cases by 2040, a 47% increase from 2020 [7]. The growing number of cancer survivors combined with the aging of populations means that survivors are living longer [8], increasing the likelihood that a significant proportion of cancer survivors will be among those affected in any disaster and reinforcing the importance of finding solutions for this subset of people.

In the wake of a disaster, regardless of type, health care is often provided by disaster shelters intended to provide a basic level of services at short notice under difficult circumstances. However, studies have shown the inadequacy of disaster shelters in terms of staffing levels and staff preparedness, access to medications, infection control, ability to handle referrals, and deficiencies in communication and mental health services, making them suboptimal in providing the type of care cancer survivors need [9].

While the evidence for the needs of cancer survivors is growing, there does not appear to be a published literature review of the impact disasters can have on cancer survivors and how to manage their maintenance care. This narrative literature review will summarize recent research about the factors important for cancer survivor care and maintaining and re-establishing care following disasters, identify knowledge gaps, and provide guidance on how to proceed before, during and following disasters.

## Methods

In this review article, we have defined the term “cancer survivor” in accordance with definitions in reports from the US National Cancer Institute and Marzorati et al. [10, 11] as: A person who continues to live and function after overcoming a serious hardship or life-threatening disease (specifically cancer) from the time of diagnosis until the end of life.

For the purposes of this narrative review, we conducted searches of the PubMed database using the relevant Index Medicus subject headings as keywords. Additionally, we performed manual searches of the reference lists of the identified articles and those related to the authors’ own body of work in the field, providing a representative and fairly complete survey of the databased literature available. While the body of literature on cancer survivorship needs and treatment disruptions is extensive, and the field of disaster medicine is growing, there are few studies at the intersection of these areas. We have discussed as many of these as practical in the text, first using a framework of care needs established by cancer survivorship research, then incorporating lessons that can be applied from studies of how disasters affect healthcare delivery in other areas. Studies that are less directly applicable to the topic but which may still offer insight are collected in a fully referenced table.

### Aspects important for improving quality of life in cancer survivors

Outpatient cancer care comprises multiple elements, including those aimed at preventing recurrence or late effects, screening for recurrence or metastases or second primary cancers, and interventions for the long-term consequences of cancer. Undertaking these elements of care often includes laboratory tests, imaging studies (including magnetic resonance imaging and mammograms), outpatient chemotherapy and radiation therapy, consultations with oncologists and other physician specialists, and outpatient symptom care. Furthermore, there are additional aspects that are important for improving quality of life in cancer survivors. These include supporting modified eating and lifestyle habits, fertility and intimacy issues, and mental health. For example, studies have shown disproportionately high rates of depression and anxiety experienced by cancer survivors [12], at roughly double the prevalence in the general population. Some cancers, including pancreatic and lung cancer and certain cancer treatments, are especially associated with depression [13].

There are some aspects of cancer care that are particularly important regarding older adult cancer survivors. Logistical and social support are also very important in this patient subset. A study by Firkins et al. [8] found that quality of life continues to be affected by cancer for many years, even decades, post-remission.

As important as patient age is in determining the needs of cancer survivors, cancer stage is equally important, as is cancer type. For example, research into the experiences of colon cancer survivors found that many of their unmet needs occur during the adjuvant treatment phase and thereafter [14].

#### Domains of needs

A systematic review by Kotronoulas et al. [15] identified eight domains of needs to be considered as part of a comprehensive care package for those living with, and after, colorectal cancers, namely physical/cognitive, psychosocial/emotional, family-related, social/societal, interpersonal/intimacy, practical/daily living, information/education, and health system/patient-clinician communication. These domains are sufficiently broad that they apply generally to all cancers, although some cancers may generate specific needs that do not fall neatly into these categories. Table 1 shows this framework enlarged as a general platform to discuss the needs of survivors across all cancers. Notably, more than half of these domains concern information, education, and healthcare provider/patient communication—all of which are easily disrupted in the event of a disaster.

**Table 1:** Individual supportive care needs of people with cancer classified into need domains

*Need domain*	*Operational definition*	*Potential effects of natural hazards*
Physical/cognitive	Need for help with symptom management of cancer-related problems, treatment-related toxicity, cognitive dysfunction	Disruption of physical care, including medication, treatment, and observation
Psychosocial/emotional	Need for help with psychological/emotional symptoms such as depressive mood, anxiety, fear/worry, despair	Additional stresses resulting from disaster, loss and disruption
Family-related and family caregiver support	Need for help with dysfunctional family relationships, fears/concerns for family future; participation of family caregivers to continue supportive care, encouragement to cancer survivors	Potential separation from family members and loss of support
Social/societal	Need for help with experience of social isolation, inefficient social support, diminished socialization	Separation from normal social connections; potential increased isolation
Interpersonal/intimacy	Need for help with altered body image or sexuality, sexual health problems, compromised intimacy with partner, loss of fertility	Exacerbation of intimacy issues resulting from stress, disruptions, or separation from partner
Practical/daily living	Need for help with transportation, will to live, out-of-hours accessibility, funeral care, financial strain, experience of restriction in daily living tasks such as housekeeping, exercise	Disruptions of many or all aspects of daily living
Information/education	Need for help with lack of information, uncertainty about diagnosis/treatment, uncertainty/lack of knowledge about selfcare	Infrastructure disruption limiting access to information
Health system/patient-clinician communication	Need for help with insufficient communication between patients and clinicians, satisfaction with care, participation in decision-making, preferences in communication	Infrastructure disruption precluding healthcare contact

Adapted from Kotronoulas et al. 2017 [13] under CC BY-NC-ND license

Duncan et al. [16] have described the non-pharmacological strategies that improve quality of life among cancer survivors, such as exercise, support groups, and stress management interventions. These included interventions in physical exercise, cognitive behavioral therapy, and mindfulness-based stress reduction, delivered face-to-face as well as online or via telephone, in addition to the provision of paper-based reading material.

In the event of a disaster, interruption of non-pharmacological support due to life disruptions, possibly including evacuation, may have an important impact on the outpatient care of cancer survivors. Considering the relationship between cancer survivors and depression that has already been shown in previous studies, it is even more important to discuss strategies to best ensure the quality of life of cancer survivors after a disaster.

## Research in disasters and cancer care

Any disaster presents immediate implications for primary health care [17]. The importance of emergency care for people directly affected by the disaster, in addition to maintaining acute care for affected populations, does not detract from the importance of maintaining long-term care for chronic diseases. A review of disaster medicine following a number of disasters reported that after devastating hurricanes in the United States in 2005, chronic conditions accounted for a third of patient visits in the days following the disaster [18]. While that study does not consider cancer patients specifically, focusing instead on chronic diabetic, renal, and cardiac patients, many of the implications apply: chronic diseases such as cancer account for one of the largest post-disaster patient populations, and these patients are at risk for worsened outcomes under such circumstances.

For patients in active cancer care, maintaining treatment is, of course, especially critical; for some treatments, delays as short as 2 days can negatively affect survival [19]. In non-disaster situations, these delays are often the result of demographic or socioeconomic disparities. However, in the setting of a disaster, when re-establishment of even the most basic healthcare services may take some days, specialized cancer care may take far longer to become available and accessible to survivors.

To further clarify the potential effects, a 2018 systematic review by Man et al. [20] identified 85 publications addressing the effect of disasters caused by natural phenomena, such as abnormal weather, on oncology services or the associated health implications for patients with cancer. This review, which primarily categorized the identified studies by disaster type, found that disasters caused by natural phenomena, such as abnormal weather, disrupted oncological care via damage to infrastructure and communication systems, disruption to medicine supply, and medical record loss. Table 2 summarizes the most notable of these studies. Among the findings of this review, the authors concluded that significant needs include the establishment or improvement of electronic medical records; the establishment of emergency plans for oncology departments; backup communication methods between healthcare providers, hospitals, and patients; structural improvements to safeguard biospecimens and important equipment; measures to minimize medication loss; and implementation of national healthcare registries as a basis for long-term follow-up studies after disasters. Notably, of the studies identified in the systemic review, the vast majority were from high-income countries, and none represented disasters in Latin America, the Caribbean, the Middle East, or Africa, highlighting a major gap in the evidence base.

**Table 2:** Summary of selected articles investigating patient care during disasters caused by natural phenomena

Study	Title	Cancer type or patient group	Disaster type	Challenges identified	Preparedness measures
Rodriguez-Rabassa et al., 2020 [21]	Impact of a natural disaster on access to care and biopsychosocial outcomes among Hispanic/Latino cancer survivors	All	Hurricane	Significant barriers to care; increased serum inflammatory cytokines	None identified
Calo et al., 2022 [22]	Disruptions in oncology care confronted by patients with gynecologic cancer following hurricanes Irma and Maria in Puerto Rico	Gynecological cancers	Hurricane	Disruptions in care; challenges in communication	Patient resilience and local community support key to resuming care
Baldwin et al., 2006 [23]	Moving hospitalized children all over the southeast: interstate transfer of pediatric patients during Hurricane Katrina	Pediatric	Hurricane	Transfer of patients overwhelmed nearby hospitals	Disaster plans must consider pediatric patients
David-West et al., 2015 [24]	Cross-sectional study of the impact of a natural disaster on the delivery of gynecologic oncology care	Gynecological cancers	Hurricane	Delays in chemotherapy, surgery; increase in loss to follow-up	Access to chemotherapy most affected by disparities in care
Dhillon et al., 2015 [25]	Hepatocellular carcinoma (HCC) outcomes in a public hospital setting: characteristics and outcomes from the Interim LSU Public Hospital (ILH) in New Orleans	Hepatocellular carcinoma	Hurricane	Lower 1-year survival	Attributed to lower use of sorafenib and procedural therapies
Grew et al., 2013 [26]	The impact of superstorm Sandy on the care of radiation oncology patients	Radiotherapy patients	Hurricane	Prolongation of radiotherapy courses; re-stimulation required in most	Additional fractions and weekend shifts added afterward to maintain total duration of therapy
Kanjanvaikoon et al., 2011 [27]	Long-term impact of natural disaster on cervical cancer demographics	Cervical cancer	Hurricane	Increased time to diagnosis and worse stage	Decreased use of screening services should be addressed as part of disaster planning
Nagasaka et al., 2014 [28]	“When you are old, have cancer and a storm is approaching” -The effects of Hurricane Sandy on cancer patients and proposals on potential interventions	All	Hurricane	Significant increase in emergency room presentation of elderly patients	House calls, early and prioritized evacuation may minimize the effects of an approaching natural disaster
Matsui et al., 2014 [29]	Ethical challenges for the design and conduct of mega-biobanking from Great East Japan Earthquake victims	All	Earthquake	1200 medical professionals left disaster area post-event	Questions whether conducting research can respond to survivors’ immediate health needs and whether truly voluntary participation can be ensured
Nakaya et al., 2015 [30]	The association between medical treatment of physical diseases and psychological distress after the Great East Japan Earthquake	All	Earthquake	Cancer treatment one of four conditions associated with psychological distress	NA
Joob et al., 2011 [31]	Lesson for management of cancerous patient in the big flooding	All	Flood	One in five cancer centers in Thailand shut down, difficulty transporting patients, potential release of radioactive material into floodwater	Establish active care teams to serve patients at home, cross-hospital referral systems, plan to import drugs in cases of shortages
Li et al., 2006 [32]	Years of potential life lost in residents affected by floods in Hunan, China	All	Flood	Higher cancer mortality in flood-affected villages	NA

Similarly, a more recent systematic review by Bell et al. [33] of 17 papers analyzing health outcomes in adults with chronic diseases (diabetes, end-stage renal disease, congestive heart failure, and chronic obstructive pulmonary disease) following earthquakes, wildfires, and hurricanes found that outcomes depended on access to care and healthcare utilization following the disaster. The authors also found conflicting evidence on whether older adults are indeed disproportionately affected by disasters, and concluded by advocating for further rigorous and standardized studies of the topic.

A review by Gorji et al. [34] of studies specific to cancer patients after disasters caused by natural phenomena and manmade disasters concluded that the post-disaster focus on providing food, water, and shelter, as well as injury treatment, to survivors led to many challenges for cancer patients. This review was notably limited in that only seven published papers on the topic were available for inclusion. Nonetheless, the review’s conclusion that health systems should specifically consider cancer patients and caregivers in preparing for disaster situations and surge capacity seems supportable.

A 2019 study by Nogueira et al. [35] examined how hurricane disruptions to active lung cancer care via radiotherapy affected survival, and found that having a declared disaster was associated with worse overall survival, and that longer-term disaster declarations were associated with worse survival. Dhillon et al. noted lower one-year survival rates (versus historic standards) among hepatocellular carcinoma patients from the interim hospital established in New Orleans in the aftermath of Hurricane Katrina, which was only able to provide limited access to hepatologist, liver transplant, and interventional radiologist services [25]. Another interesting study found that after Hurricane Maria in 2017, cancer survivors had reduced access to care compared with non-cancer participants. Additionally, all participants had reduced psychosocial well-being on psychosocial questionnaires, and showed increased serum inflammatory cytokines [21].

For cancer survivors themselves, there are actions for long-term and short-term preparations that can be taken in advance of a disaster. Some of these steps have been proposed by the Centers for Disease Control, National Cancer Institute, and American Cancer Society, among others [36–38]. Among other suggestions, these sources recommend preparing a disaster plan with family and care providers; assembling an emergency supply kit that includes medical supplies, insurance and clinical trial information, and a copy of the patient’s survivorship care plan; locating nearby disaster shelter locations; and taking steps to avoid potentially dangerous infections that may be prevalent in a post-disaster setting. These procedures ask cancer survivors to take action to prepare themselves for disasters; therefore, cancer survivors themselves must decide whether, how, and when to do so. Cancer care providers should ensure that cancer survivors are aware of these recommendations and plan ahead for the possibility of a disaster.

### Climate change and disasters

Disasters caused by extreme weather events associated with climate change can adversely affect the treatment and care of vulnerable populations, including cancer survivors and those with specific medical needs. Recently, because of global warming, heavy rains have occurred more frequently, tropical cyclones have become increasingly stronger [39], and the speed of tropical cyclones has slowed [40]. As global warming progresses, disasters related to climate change can be expected to occur with increasing regularity and become more severe. The number of people over the age of 65 years is also increasing worldwide, as is overall life expectancy [41]; as a result, the number of cancer survivors continues to grow [42]. While the numbers of cancer survivors of all ages are increasing, older adult cancer survivors have underlying diseases other than cancer and require complex medical interventions. With the convergence of the trend in extreme weather and the trend in number of cancer survivors, emergency preparedness measures for cancer survivors are increasingly urgent issues in the context of climate change.

The published studies to date, or the lack thereof, indicate that there are many areas that remain poorly studied or entirely unstudied. While this is understandable given the difficulty in collecting data in an accurate and rigorous manner considering the chaos and resource restrictions after a disaster, the need for further research is clearly established, and suggestions for further directions in research are, therefore, described here.

## Future directions

Despite the relative dearth of systematic research into how disasters affect cancer care, some studies have clearly identified some potential solutions that could be implemented. Approaches to these problems may be broadly generalized into the categories of technological solutions and improved planning.

### Technological solutions

Technological evolution promises a number of new areas where improvements could be made in disaster care for cancer survivors. For example, adaptations in response to the COVID-19 pandemic have resulted in numerous new approaches and advances in telemedical care. Telemedicine was found to provide support and connectedness in the majority of advanced cancer patients receiving palliative care a hospital in eastern India during the pandemic [43]. In another longitudinal study conducted in Italy, patients (including those with cancer) receiving palliative care used telemedicine for therapy modifications during the outbreak of COVID-19 and the majority of patients declared that they would continue using telemedicine after the pandemic [44]. With advance planning, this type of telemedicine approach could provide a basis for continuation of some elements of long-term care in the case of a disaster.

However, communication infrastructure is often unreliable after certain types of disaster, potentially limiting the application of telemedicine-based approaches in all cases. Although new technological advances, not yet available in all areas, make it less likely that disasters will disrupt internet, telephone or data service, it is important for cancer survivors to be able to manage, rapidly retrieve, and use information on how to continue home care and treatment during normal times.

Self-management of one’s medical information and self-disclosure in a crisis situation are therefore particularly useful for prompt treatment, nursing, and transportation by medical teams who may have come from outside to assist in the event of a disaster. One approach to addressing this need is exemplified by a smartphone application and accompanying web-based application developed by Nakai et al. [45], which records medical information on a user’s smartphone that, in a disaster, may be disclosed to medical experts (at the user’s discretion) in requesting assistance in meeting needs. This information allows plotting of their locations and other information, such as necessary supplies, in a hypothetical disaster. This simple mechanism has the potential to help patients continue their care even if the communication infrastructure is unavailable [45]. However, this system remains under development and only a Japanese version is available at this time; it also relies on the availability of smartphone technology among users and continued service in a disaster, but it shows the potential for information-sharing technologies during such a situation.

Relatedly, while a growing body of evidence has shown the benefit of electronic health records versus paper records in patient care, disasters are often accompanied by power failures or damage to facilities that can impede their use in disaster situations. Alternative methods of accessing or storing medical records must therefore be considered to enable continuity of care where possible. These include redundancy and the remote or offsite storage of medical records, as well as planning for backup options in the case of disaster, as exemplified in a case study from a New York City-area hospital that was completely flooded during Hurricane Sandy in 2012 [46].

The difficulties in maintaining electronic health records in a disaster highlight the importance of information-sharing technologies. To this end, application of epidemiology methods within disaster management processes may be particularly apt [47], especially the importance of disaster registries for cancer survivors as a population of special need.

For their part, cancer survivors themselves should manage their own up-to-date care information and update it regularly. At the same time, hard copies of regularly updated printed information should be maintained in case electronic medical records are damaged or difficult to access in the event of a disaster. This method, although primitive, may be an effective solution for obtaining appropriate treatment and care during disasters.

### Planning and preparation

Beyond the promise offered by technology, the importance of disaster planning for cancer survivor care, with important steps to be taken before, during, and after a disaster, cannot be overstated.

Planning for continuation of cancer care services in the setting of disasters must begin with the identification of cancer survivors as a vulnerable population requiring attention and assistance during a disaster. This will require the application of local emergency preparedness guidelines and planning for patient prioritization in accordance with those guidelines, as well as coordination across different levels of government [48]. A white paper by Gebbie et al. [49] offers guidance on adapting standards of care in acute emergency situations.

While this guidance information is provided unilaterally for cancer survivors to prepare for disasters and be self-reliant, cancer survivors must actively participate in the process of planning for the continuation of cancer care services in the event of a disaster.

The ongoing COVID-19 pandemic presents unprecedented complications for disaster medicine. For example, cancer survivors undergoing chemotherapy have weakened immunity, making it difficult to judge whether it is appropriate to evacuate to a shelter where viral transmission may be more likely during a COVID-19 outbreak. Evacuation to a hospital is an option during a disaster, but if the hospital is contaminated with COVID-19, this may not be the best option. Research on (and planning for) such situations must be considered as the pandemic continues. Lessons learned from the COVID-19 pandemic (in addition to the pandemic-inspired innovations in telemedicine noted above) may prove invaluable in preparing for future disasters.

In recent years, smartphones have become the top of the list of items that must be brought with you when evacuating. In a survey of Japanese elementary school and junior high school students, smartphones were the most-identified resource to be sure to bring when evacuating, followed by a wallet [50]. Future research on disaster preparedness and information management using smartphones and other wearable devices will be more important not only for cancer survivors but also for anyone receiving outpatient treatment.

Identification of how cancer survivors manage their own medical information and treatment information in preparation for evacuation from disaster-affected areas is necessary to further accumulate knowledge on mechanisms that can efficiently monitor and utilize information. Quantification and evaluation of these methods will be necessary to determine which are effective under disaster circumstances and which are too technologically burdensome for current cancer patients and long-term survivors.

### Knowledge gaps

Beyond these potential and evolving areas for improvement, there is much that remains unknown and must be addressed before solutions can be proposed. Further research is needed to address these gaps in the literature regarding cancer care disruptions after disasters caused by natural phenomena. There currently exist gaps in both the existing research and barriers to implementation of identified strategies.

In terms of gaps in the existing research, an immediate requirement is studies with longer follow-up periods, as studies to date have been limited in this aspect, largely focusing instead on the immediate aftermath of the disaster. More studies, such as the long-term retrospective by Ozaki et al. [4], will be invaluable and data should be collected to enable similar studies to be performed in the future. Relatedly, because most studies to date have only examined clinical endpoints, future studies should strongly consider the collection of quality-of-life (QOL) endpoints, such as via validated QOL instruments.

Survivors of different types of cancers may have differing long-term needs; thus, studies of a particular cancer type will not be universally applicable. Added to this is the need for further evaluation specifically in aging populations, which is especially pressing with cancer survivors living longer. There is also a notable lack of research from low- and middle-income settings, which may be the least-prepared and most affected populations in the event of a disaster. As an initial preliminary step toward bridging these gaps, healthcare planning for disasters should incorporate plans for the systematic collection of data afterward. This forward-looking approach should help ensure that future plans for cancer care management in the setting of a disaster have a firm and scientifically valid foundation.

In addition to research to be addressed in the future, there remain gaps in implementation of the lessons already learned via existing research. These can be generally grouped into technological implementation, planning gaps, and funding gaps. Technological implementation will require bolstering of electrical power and communications infrastructure, as well as improving the remote storage of medical records and treatment plans. The promise of telemedicine has been significantly advanced and validated in the global response to the COVID-19 pandemic, yet it remains to be ensured that those lessons gained from the pandemic are applied to disaster planning. These planning gaps must be bridged by attaining buy-in from multiple levels of government, and by using this coordinated approach to ensure standardization across settings. Lastly, funding gaps are likely to remain an issue as disaster planning remains underfunded in many, if not most, areas. Technological development requires investment, as does the availability of funds for post-disaster response. It is incumbent upon healthcare providers, researchers, cancer survivorship advocates, governments and societies to advocate for and ensure the provisions of these funds in anticipation of future disasters.

## Conclusions

The increasing numbers of cancer survivors and survivor longevity, combined with increases in the number of disasters caused by natural phenomena, means that as a society, we must address the issue of maintaining quality of life and care for cancer survivors experiencing disruptions associated with disasters caused by natural phenomena. The research to date in disaster-related disruptions of the provision of care to cancer survivors shows how treatment gaps negatively affect outcomes, and quality of life can be significantly affected as well. There are likely to be variations of these effects based on settings, resources available, and patient groups affected by the disaster. However, there are unlikely to be any groups or situations in which a disaster has minimal negative effects versus those of the general population. Overall, the body of research has shown that potential solutions are available with sufficient forethought and careful planning, although there are significant gaps in research and barriers to implementation of these solutions.

Various public agencies have provided recommendations for cancer survivors to prepare for a disaster, but the burden is upon the cancer survivors themselves to be aware of and implement these recommendations. Research is needed on solutions that help ensure that cancer survivors are prepared. For example, entering their information in a smartphone app and using it to manage their care during normal times may make it possible to refer to the temporary information remaining on the smartphone in the event that communication infrastructure is disrupted. Therefore, clarifying how cancer survivors manage their own medical and treatment information is necessary to further accumulate knowledge about mechanisms for efficiently monitoring and utilizing information.

The gaps in this research necessitate forward-looking planning for disaster care research that must consider adaptations of study designs, including long-term follow-up and QOL endpoints and the inclusion of varying and under-studied subgroups. Approaches to filling these gaps must include recognition of the implementation gaps as well, which will require technological innovation, coordinated and standardized planning across levels of government, and sufficient funding. For institutions providing long-term survivorship care, it will be particularly important to be aware of all of these gaps and to undertake definitive and proactive steps to overcome them.

Clinicians, caregivers, institutions, and organizations should work toward conducting research, developing disaster plans, improving processes to be sustainable in the event of a disaster, and preserving healthcare resources for a growing number of cancer survivors.

In advance of any potential disaster, plans for medical resource protection and support for cancer survivors should be prepared based on their current medical condition. Standardized disaster countermeasures have the potential risk of mismatching supply and demand during disasters. Therefore, it is necessary to protect medical resources based on the psychosomatic condition of each cancer survivor.

To that end, the effective utilization of information technology has become increasingly important. However, in preparation for potential disruptions of the power supply and communication infrastructures that often arise following disasters caused by natural phenomena, there is a need for a mechanism that seamlessly allows for traditional paper backup of information in parallel with the use of information technology. Research on the implementation of these solutions is lacking.

During the course of disasters caused by natural phenomena and their immediate aftermath, disaster care measures may well be focused on establishing trauma care and emergency services, depending on the type of disaster. However, authorities must be prepared for the maintenance or timely re-establishment of cancer care services as well. Furthermore, it is most important for cancer survivors to be prepared to seek help at any time and to be willing to disclose their medical information to available specialists. This applies of course to all vulnerable populations, not just cancer survivors, but the continuity of care is important for this population, with care disruptions leading to worse outcomes.

After a disaster, when healthcare services have been restored and the danger has passed, attention should be paid to understanding what happened in terms of the effects on cancer survivors via well-conducted scientific research aimed at filling the gaps in our understanding.

## Data Availability

All data generated or analyzed during this study are included in this published article.
